# Impact of preoperative malperfusion on postoperative outcomes in type A aortic dissection - importance of serum lactate estimation in ongoing malperfusion

**DOI:** 10.1177/02676591231157545

**Published:** 2023-02-16

**Authors:** Tim Kaufeld, Erik Beckmann, Linda Rudolph, Heike Krüger, Ruslan Natanov, Morsi Arar, Wilhelm Korte, Jessica Kaufeld, Tobias Schilling, Axel Haverich, Malakh Shrestha, Andreas Martens

**Affiliations:** 1 Department of Cardiothoracic, Transplant and Vascular Surgery, 9177 Hannover Medical School, Hannover, Germany; 2 Minneapolis Heart Institute, Abbott Northwestern Hospital, United States of America; 3 Department of Nephrology and Hypertension, 9177 Hannover Medical School, Hannover, Germany; 4 Cardiovascular Medicine, Mayo Clinic, Rochester, MN, USA

**Keywords:** perfusion, malperfusion, Acute type A aortic dissection, aortic dissection, aortic repair, aortic surgery, arch repair, surgical treatment

## Abstract

**Introduction:**

Acute type A aortic dissection (ATAAD) is one of the most critical emergencies in cardiovascular surgery. Additional complications such as organ malperfusion can significantly decrease the chances of survival. Despite promptly performed surgical treatment, impaired organ perfusion may persist, thus close postoperative monitoring is recommended. But, is there a surgical consequence due to the existence of a preoperatively known malperfusion and is there a correlation between pre-, peri- and postoperative levels of serum lactate and proven malperfusion?

**Methods:**

Between 2011 and 2018, 200 patients (66% male; median age: 62.5 years; interquartile range: +/−12.4 years) that received surgical treatment at our institution for an acute dissection DeBakey type I were enrolled in this study. The cohort was divided into two groups according to the preoperative existence of malperfusion and non-malperfusion. At least one kind of malperfusion occurred in 74 patients (Group A: 37%), while 126 patients (Group B: 63%) showed no evidence of malperfusion. Furthermore, lactate levels of both cohorts were differentiated into four periods: preoperative, intraoperative, 24 hours after surgery, and 2–4 days after surgery.

**Results:**

The patients’ status differed significantly prior to surgery. Group A (malperfusion) showed an elevated requirement for mechanical resuscitation (A: 10.8%; B: 5.6%; *p*: 0.173), were significantly more often admitted in an intubated state (A: 14.9%; B: 2.4%; *p*: 0.001) and showed higher incidences of stroke (A: 18.9% (*n* = 149); B: 3.2% (*n* = 4); *p*: 0.001). Levels of serum lactate from the preoperative period until days 2–4 were significantly increased in the malperfusion cohort at all times.

**Conclusions:**

Preexisting malperfusion due to ATAAD may significantly increase the chance of early mortality in patients with ATAAD. Serum lactate levels were a reliable marker for inadequate perfusion from admission until day 4 after surgery. Despite this, early intervention survival in this cohort remains limited.

## Introduction

**Figure 1.**
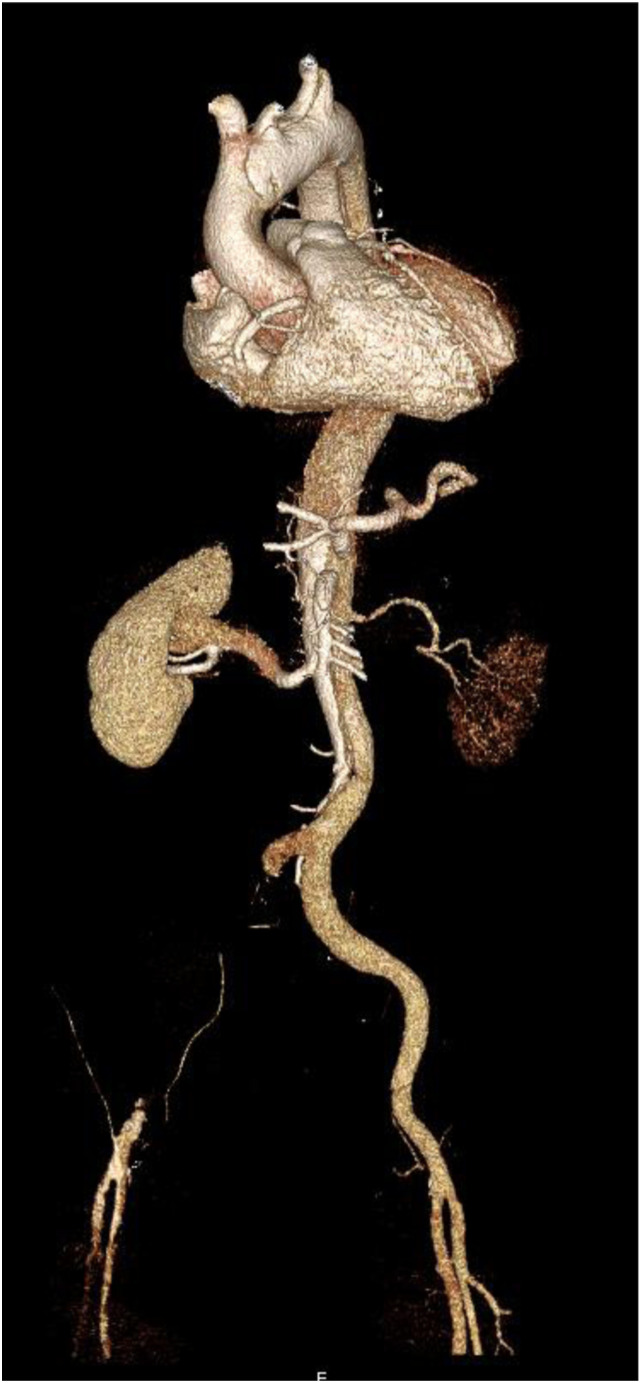
3D CTA reconstruction; ATAAD with malperfusion left renal artery and right common iliac artery.

Acute type A aortic dissection (ATAAD) is one of the most critical emergencies in cardiovascular surgery. The diagnosis of ATAAD requires clinical indications as well as radiographic evidence of an aortic dissection. An intimal tear in the ascending aorta extends into the aortic wall media to create a false lumen and a dissection flap. This can result in a severe decrease in organ perfusion (Figure 1). Even without peripheral malperfusion, ATAAD remains a life-threatening disease. Additional complications such as organ malperfusion can significantly decrease the chance of survival. Due to this fact, the rapid restoration of flow into the true lumen is necessary and surgical aortic repair remains the gold standard. However, clinical signs of the extent of a compromised perfusion are typically not well presented at later stages. Nevertheless, despite promptly performed surgical treatment, organ malperfusion may persist, thus postoperative monitoring should be closely performed. Therefore, reliable biomarkers such as serum lactate have been suggested as potential indicators for detecting persistent ischemia and increased early mortality.^1–4^

We evaluated the surgical consequences resulting from the existence of a preoperatively known malperfusion. In addition, pre- and postoperative levels of serum lactate were verified for a possible correlation with proven malperfusion.

## Methods

### Definitions

Clinical signs, including abdominal or back pain, were observed, and signs of malperfusion or neurological symptoms may also occur as unspecific symptoms. The radiographic equivalent of an ATAAD was either the existence of a dissection membrane starting in the ascending aorta or an intramural hematoma inside the aortic wall. Malperfusion was defined as an occlusion or a false lumen perfusion of one relevant artery per organ. We characterize end-organ malperfusion according to imaging findings and clinical signs of malperfusion. Furthermore, the diagnosis of malperfusion was defined according to Sievers et al.^5^ M2: Dissection of at least 1 supra-aortic vessel or aortic arch true lumen collapse with (M2+) or without (M2-) clinical symptoms of cerebral (stroke) or upper extremity (pulse deficit, pain, pallor, paraesthesia) malperfusion; M3: dissection or false lumen origin of at least 1 visceral, renal or 1 iliac artery or aortic true lumen collapse entailing functional closure of at least 1 visceral, renal or iliac artery offspring, with (M3+) or without (M3-) clinical symptoms of bowel (abdominal pain, ileus, bloody diarrhoea), kidney (anuria, renal failure signs) or lower extremity (pulse deficit, pain, pallor, paraesthesia) ischaemia.^5^ Therefore, patients with stages M2 and M3

((−), (+)) were assigned to the malperfusion group. The presence of extension of the aortic dissection, the entry location and the dissection of the aorta, supra-aortic, visceral and iliac arteries were analysed according to a CTA. To differentiate lactate levels according to the anatomic location we further distinguished the cohort into cerebral, visceral, renal, and limb malperfusion. Furthermore, lactate levels of both cohorts were differentiated into four periods: preoperative, intraoperative, 24 hours after surgery, and 2–4 s after surgery. During these time periods, the highest value of serum lactate was recorded for each period.

Patients with severe neurologic symptoms at admission, like hemiplegia, dysarthria, or apraxia, without a performed cerebral CT scan and with postoperative evidence of stroke were assigned to the preoperative stroke cohort. Postoperative stroke had to be confirmed by magnetic resonance imaging or computed tomography. Accidently induced ATAAD during open heart surgery is referred to as iatrogenic dissection. Dissections of coronary arteries were either preoperatively discovered during coronary angiography or intraoperatively visible.

### Study population and study design

Individual consent was obtained from all patients to allow for further follow-up examinations. In this study, we included 200 patients (66% male; median age: 62.5 years; interquartile range: +/−12.4 years) that received surgical treatment at our institution between 2011 and 2018 due to an ATAAD. DeBakey type II + III dissections as well as chronic dissections were not included.

The subjects were divided in two cohorts according to the preoperative existence of malperfusion and non-malperfusion. As per the definition,^5^ at least one kind of malperfusion occurred in 74 patients (Group A: 37%), while 126 patients (Group B: 63%) showed no evidence of malperfusion. Data correlates with the fact that one patient can present different kinds of malperfusion. However, patients were actively contacted by a study nurse team. Furthermore, data were collected contemporaneously in our outpatient clinic. The data were also reviewed retrospectively and supplemented from the patients’ records after informed consent. This retrospective study was approved by our institutional ethics committee. Preoperative characteristics of the evaluated cohort are presented in Table 1 and 2.

**Table 1.** Patients characteristics.

Characteristics	Entire cohort	Pre operative malperfusion	Non pre operative malperfusion	*p*-value
Total patients	*n* = 200	*n* = 74	*n* = 126	
Age at surgery (years), mean ± SD	62.5 ± 12.3	61.1 ± 13.6	63.3 ± 11.5	.232
Sex male, *n* (%)	131 (65.5)	49 (66.2)	82 (65.1)	.870
BMI, median (IQR)	26.3 (24.0–29.2)	26.4 (23.8–28.8)	26.2 (24.5–29.2)	.789
Hypertension, *n* (%)	119 (59.5)	44 (59.5)	75 (59.5)	.993
Diabetes mellitus, *n* (%)	12 (6.0)	3 (4.1)	9 (7.1)	.541
Pvod, *n* (%)	6 (3.0)	4 (5.4)	2 (1.6)	.197
COPD, *n* (%)	13 (6.5)	8 (10.8)	5 (4.0)	.075
Coronary heart disease, *n* (%)	21 (10.5)	6 (8.1)	15 (11.9)	.398
Hyperthyreosis, *n* (%)	0 (0.0)	0 (0.0)	0 (0.0)	
Hypothyreosis, *n* (%)	16 (8.0)	6 (8.1)	10 (7.9)	.966
Atrial finrillation, *n* (%)	25 (12.5)	8 (10.8)	17 (13.5)	.580
Marfan syndrom, *n* (%)	7 (3.5)	3 (4.1)	4 (3.2)	.711
Bicuspid aortic valve, *n* (%)	14 (7.0)	6 (8.1)	8 (6.3)	.638
Pericardial tamponade, *n* (%)	61 (30.5)	21 (28.4)	40 (31.7)	.617
Preoperative intubation, *n* (%)	14 (7.0)	11 (14.9)	3 (2.4)	**.001**
Mechanical resuscitation, *n* (%)	15 (7.5)	8 (10.8)	7 (5.6)	.173
Cardiac-reoperation, *n* (%)	3 (1.5)	2 (2.7)	1 (0.8)	.556

IQR: interquartile range; BMI: body mass index; PVOD: peripheral vascular; COPD: chronic obstructive occlusion disease.

**Table 2.** Preoperative data.

Characteristics	Entire cohort	Pre operative malperfusion	Non pre operative malperfusion	*p*-value
Total patients	*n* = 200	*n* = 74	*n* = 126	
Cerebral malperfusion, *n* (%)	36 (18.0)	36 (48.6)	0 (0.0)	**<.001**
Visceral malperfusion, *n* (%)	17 (8.5)	17 (23.0)	0 (0.0)	**<.001**
Limb malperfusion, *n* (%)	26 (13.0)	26 (35.1)	0 (0.0)	**<.001**
Renal malperfusion, *n* (%)	25 (12.5)	25 (33.8)	0 (0.0)	**<.001**
Hemiparesis, *n* (%)	9 (4.5)	9 (12.2)	0 (0.0)	**<.001**
Paraparesis, *n* (%)	5 (2.5)	5 (6.8)	0 (0.0)	**.006**
Seizure, *n* (%)	1 (0.5)	0 (0.0)	1 (0.8)	1.000
Evidence of stroke CT, *n* (%)	18 (9.0)	14 (18.9)	4 (3.2)	**<.001**
Neurologic symptoms, *n* (%)	46 (23.0)	41 (55.4)	5 (4.0)	**<.001**
Dissection supra-aortic arteries, *n* (%)	58 (29.0)	43 (58.1)	15 (11.9)	**<.001**
Dissection LCA, *n* (%)	5 (2.5)	3 (4.1)	2 (1.6)	.361
Dissection RCA, *n* (%)	20 (10.0)	11 (14.9)	9 (7.1)	.079
Iatrogenic dissection, *n* (%)	5 (2.5)	2 (2.7)	3 (2.4)	1.000
Onset of pain to surgery time (h), median (IQR)	5.0 (3.6–12.0)	4.0 (3.0–8.0)	7.0 (4.0–16.0)	**.001**

CT: computer tomography; LCA: left coronary artery; RCA: right coronary artery; IQR: interquartile range.

### Follow-up

We obtained informed consent from patients to allow the collection of follow-up data. The clinical follow-up ended and was completed in November 2021. Patients were seen frequently in our aortic outpatient clinic. Imaging via magnetic resonance or computed tomography angiography was performed regularly after surgery and during follow-up.

### Perioperative management and surgical technique

According to our standard protocol, all patients with acute ATAAD were transferred to the operation theater directly after first diagnosis. To avoid decompensation, intubation was not performed before anesthetic and surgical preparations were completed. After median sternotomy, extracorporeal circulation (ECC) was established. Our cannulation technique of the ascending aorta, also in ATAAD, was previously published by our group.^6,7^ We prefer a direct cannulation of the ascending aorta after identifying the true lumen using transesophageal echocardiography. The left side of the heart was vented through the right superior pulmonary vein. The aorta was clamped, also in ATAAD patients. In all dissections, cardioplegia was administered directly into the coronary ostia. Blood cardioplegia was our preferred method of myocardial protection. According our root-first policy we performed root/ascending aortic repair while cooling the patient to a nasopharyngeal temperature of 22–26°C. Other concomitant procedures (e.g., CABG) were performed if necessary. Cardioplegia was repeated approximately every 30 min.^7^ Furthermore, in patients with extended arch repair we implemented a beating-heart technique in 2010.^8^ In all cases, either a proximal, subtotal (involving replacement of the brachiocephalic trunk), or total arch replacement with ET or FET, hypothermic circulatory arrest (temperatures between 22°C and 26°C) and bilateral selective antegrade cerebral perfusion was performed. The application of SACP varied when a limited arch repair was performed.

### Extended arch repair

From 2000–2010, the FET technique was performed using a custom-made Chavan-Haverich prosthesis followed by a prefabricated Chavan-Haverich hybrid graft^9,10^ (Curative GmbH, Dresden, Germany). From 2005 until 2010, the Jotec E-vita hybrid graft was used.^11^ Supra-aortic vessels were reattached using the island technique (*en bloc*) until 2010. In contrast to the *en bloc method* the four-branched frozen elephant trunk prosthesis (FET Vascutek Terumo, Terumo®, Glasgow, UK) was established, which we have used continuously since 2010.^12,13^ For total or hemi-arch replacement we changed our strategy in 2007 from a straight graft with island technique to a branched Sienna™ graft (Terumo®, Glasgow, UK). The extensive use of the branched aortic arch prosthesis resulted in major technical changes. As a consequence of these changes, arch replacement was performed after completing cardiac and distal aortic repair. Head vessels were anastomosed to the corresponding side branches of the graft at the end of the procedure.^12^

### Proximal arch repair

An isolated replacement of the proximal aortic arch was performed using established straight Dacron grafts.

### Statistical analysis

SPSS 27 Statistics software (IBM Corp. released 2020; IBM SPSS Statistics for Windows, Version 27.0; Armonk, NY: IBM Corp.) was used for the data analysis. A normal distribution of variables was calculated using the Kolmogorov–Smirnov test. Categorical variables are stated as absolute numbers (*n*) and proportions. Normally distributed continuous variables are stated as mean ± standard deviation, while continuous variables without normal distribution are stated as median and interquartile range (IQR). Fisher’s exact test was used to detect differences in categorical variables. Differences in continuous variables were tested using the Man- Whitney *U* test. Kaplan-Meier analysis and log rank were used for the evaluation of survival, and the log rank test was used to test for differences. We did not correct for multiple testing. A value of *p* < 0.05 was considered statistically significant.

## Results

### Preoperative patient characteristics

Preoperative patient characteristics showed no significant difference regarding age (A: 61.1 y; B: 63.3 y; *p*: 0.232). The majority in both cohorts were male patients (A: 66.2%; B: 65.1%; *p*: 0.870). Diseases like COPD (A: 10.8%; B: 4.0%; *p*: 0.197) and PVOD (A: 5.4%; B: 4.0%; *p*: 0.197) were higher but not significantly in the malperfusion group. Furthermore, the data proved a prevalence of 59.5% (*p*: 0.993) of patients in each cohort for hypertension. Other concomitant diseases occurred without significant differences. The hemodynamic conditions of both groups differed at admission. Patients in Group A (malperfusion) showed an elevated demand for mechanical resuscitation due to pulseless electrical activity (A: 10.8%; B: 5.6%; *p*: 0.173) and were significantly more often admitted to the hospital in an intubated status than the group without malperfusion (A: 14.9%; B: 2.4%; *p*: 0.001). Group A was composed as follows: cerebral malperfusion *n* = 36 (48.6%), visceral malperfusion *n* = 17 (23.0%), limb malperfusion *n* = 26 (35.1%), and renal malperfusion *n* = 25 (33.8%). In keeping with an increased rate of dissection of the supra-aortic arteries (A: *n* = 43 (58.1%); B: *n* = 15 (11.9%); *p*: 0.001) the malperfusion group showed a higher incidence of stroke (A: *n* = 14 (18.9%); B: *n* = 4 (3.2%); *p*: 0.001) and preoperative neurological symptoms (A: *n* = 41 (55.4%); B: *n* = 5 (4.0%); *p* 0.001). Interestingly, the onset of pain to surgery time was significantly reduced in Group A (A: 4.0 h; B: 7.0 h; *p*: 0.001).

### Intraoperative data

Intraoperative data are shown in Table 3. Despite comparable operation times (A: 379.7 min; B: 363.1 min; *p*: 0.281), cardiopulmonary bypass times (A: 257.7 min; B: 244.7 min; *p*: 0.263), aortic cross-clamp times (A = 130.2 min vs. B = 132.2 min; *p* 0.789), duration of hypothermic circulation (A: 42.5 min; B: 34.0 min; *p*: 0.011), and SACP (A: 76 min; B: 51.5 min; *p*: 0.070), times were longer in the malperfusion group. Root involvement was more frequently presented in Group B (A: *n* = 35 (47%); B: *n* = 79 (62.7%); *p*: 0.034). In keeping with this result, the number of David procedures was increased in Group B (A: *n* = 12 (16.2%); B: *n* = 34 (27.0%); *p*: 0.081). Furthermore, patients with evidence of malperfusion received less ECMO treatment (A: *n* = 3 (4.1%); B: *n* = 11 (8.7%); *p*: 0.211).

**Table 3.** Intraoperative data.

Characteristics	Entire cohort	Pre operative malperfusion	Non pre operative malperfusion	*p*-value
Total patients	*n* = 200	*n* = 74	*n* = 126	
Total operation time (min), mean ± SD	369.3 ± 105.2	379.7 ± 103.8	363.1 ± 105.9	.281
Cardiopulmonary bypass time (min), mean ± SD	249.5 ± 79.3	257.7 ± 81.8	244.7 ± 77.8	.263
Aortic cross-clamp time (min), mean ± SD	131.5 ± 52.6	130.2 ± 48.0	132.3 ± 55.3	.789
HCA (hypothermic circulatory arrest) time (min), median (IQR)	37.0 (22.3–54.8)	42.5 (29.8–58.5)	34.0 (20.0–52.0)	**.011**
SACP (selective antegrade cerebral perfusion) time (min), median (IQR)	60.0 (20.0–94.8)	76.0 (28.5–96.5)	51.5 (19.0–93.3)	.070
Minimal core temperature (°C), median (IQR)	25.0 (22.8–26.0)	24.8 (22.0–26.0)	25.0 (23.0–26.0)	.585
Erythrocyte concentrates, median (IQR)	6.0 (4.0–9.0)	6.0 (4.0–10.0)	5.0 (3.0–8.0)	**.034**
Fresh frozen plasma, median (IQR)	6.0 (4.0–6.8)	6.0 (4.0–8.0)	6.0 (4.0–6.0)	.068
Platelet concentrates, median (IQR)	3.0 (2.0–4.0)	3.5 (2.0–4.0)	3.0 (2.0–4.0)	.499
Beating heart, *n* (%)	63 (31.5)	27 (36.5)	36 (28.6)	.245
Proximal arch replacement, *n* (%)	78 (39.0)	26 (35.1)	52 (41.3)	.390
Subtotal arch replacement, *n* (%)	18 (9.0)	6 (8.1)	12 (9.5)	.736
Total arch replacement, *n* (%)	8 (4.0)	2 (2.7)	6 (4.8)	.713
Total arch replacement elephant trunk, *n* (%)	9 (4.5)	6 (8.1)	3 (2.4)	.079
Total arch replacement frozen elephant trunk, *n* (%)	87 (43.5)	34 (45.9)	53 (42.1)	.593
Bio glue, *n* (%)	76 (38.0)	33 (44.6)	43 (34.1)	.141
Aortic valve replacement bio, *n* (%)	33 (16.5)	13 (17.6)	20 (15.9)	.755
Aortic valve replacement mech, *n* (%)	26 (13.0)	8 (10.8)	18 (14.3)	.480
Root involvement, *n* (%)	114 (57.0)	35 (47.3)	79 (62.7)	**.034**
Bentall, *n* (%)	58 (29.0)	21 (28.4)	37 (29.4)	.882
David, *n* (%)	46 (23.0)	12 (16.2)	34 (27.0)	.081
Yacoub, *n* (%)	2 (1.0)	0 (0.0)	2 (1.6)	.531
CABG, *n* (%)	39 (19.5)	13 (17.6)	26 (20.6)	.597
ECMO, *n* (%)	14 (7.0)	3 (4.1)	11 (8.7)	.211
Exitus in tabula, *n* (%)	0 (0.0)	0 (0.0)	0 (0.0)	

IQR: interquartile range; min: Minute; SD: standard deviation; HCA: hypothermic circulatory arrest time; SACP: selective antegrade cerebral perfusion time; CABG: coronary artery bypass graft.

### Postoperative data

The postoperative data are presented in Table 4. Survival time (A: 169.0 d (11.8–1686.0); B: 1667.5 d (718.3–2537.0); *p*: < .001) and 30 day mortality (A: 35.1% (*n* = 26); B: 11.1% (*n* = 14); *p*: < .001) differ significantly between the groups. In addition, median ventilation time (A: 89.0 h (34.5–208.3); B: 39.0 h (25.0–98.0); *p*: 0.001) and median stay in the ICU (A: 5.0 d (3.0–10.0); B: 4.0 d (2.0–7.0); *p*: 0.048) were prolonged in the malperfusion group. Postoperative dialysis was also required significantly more often in Group A (A: 29.7% (*n* = 22); B: 7.9% (*n* = 10); *p*: < .001). Despite the fact that the total incidence of stroke was increased in the malperfusion group (A: *n* = 32 (43.2%); B: *n* = 21 (16.7%); *p*: < .001), new-onset postoperative stroke did not differ between the groups (A *n* = 9 (12.2%); B: *n* = 16 (12.7%); *p*: 0.912). New-onset (postoperative) dissection-related malperfusion occurred in 11 patients with cerebral malperfusion and three patients with new renal malperfusion in comparison to the initially (preoperative) performed CTA.

**Table 4.** Postoperative data.

Characteristics	Entire cohort	Pre operative malperfusion	Non pre operative malperfusion	*p*-value
Total patients	*n* = 200	*n* = 74	*n* = 126	
Survival time (days), median (IQR)	1386.5 (103.5–2251.5)	169.0 (11.8–1686.0)	1667.5 (718.3–2537.0)	**<.001**
Ventilation time (h), median (IQR)	51.5 (25.0–138.0)	89.0 (34.5–208.3)	39.0 (25.0–98.0)	**.001**
Intensive care unit (days), median (IQR)	5.0 (3.0–8.0)	5.0 (3.0–10.0)	4.0 (2.0–7.0)	**.048**
Rethoracotomy, *n* (%)	25 (12.5)	12 (16.2)	13 (10.3)	.223
Dialysis, *n* (%)	32 (16.0)	22 (29.7)	10 (7.9)	**<.001**
30 day mortality, *n* (%)	40 (20.0)	26 (35.1)	14 (11.1)	**<.001**
CCT stroke, *n* (%)	53 (26.5)	32 (43.2)	21 (16.7)	**<.001**
New-onset stroke, *n* (%)	25 (12.5)	9 (12.2)	16 (12.7)	.912
Persisting cerebral malperfusion, *n* (%)	14 (7.0)	3 (4.1)	11 (8.7)	.211
Persisting limb malperfusion, *n* (%)	4 (2.0)	4 (5.4)	0 (0.0)	**.018**
Persisting visceral malperfusion, *n* (%)	8 (4.0)	8 (10.8)	0 (0.0)	**<.001**
Persisting renal malperfusion, *n* (%)	15 (7.5)	12 (16.2)	3 (2.4)	**<.001**

IQR: interquartile range; min: Minute; SD: standard deviation; CCT: cranial computertomography.

### Long-term follow-up data

**Figure 2.**
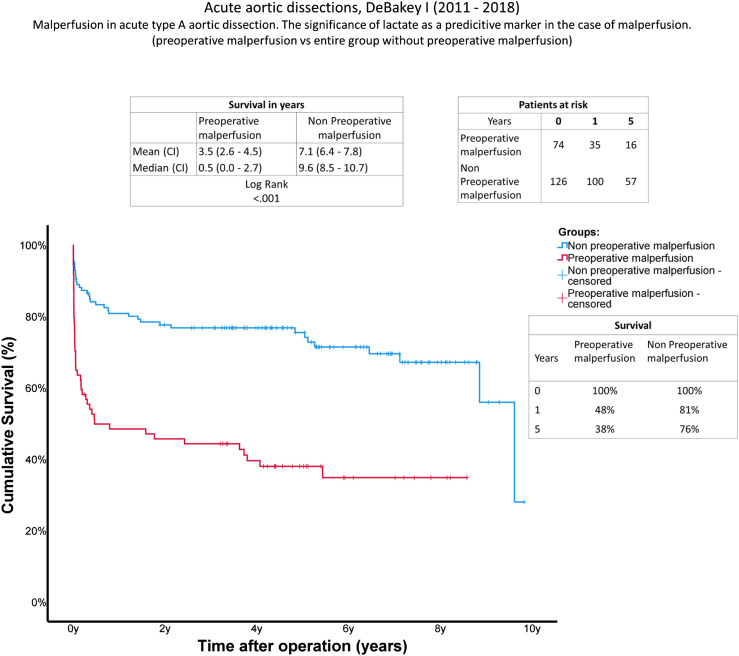
Survival: Kaplan-Meier curves showing survival with and without preoperative cardiac tamponade. The *x*-axis denotes the time after operation.

Follow-up data can be found in Table 5. During follow-up, the rate of aortic reintervention was 8.0% in total. Secondary aortic surgery was increased in patients of Group A at 13.5% (A: 13.5% (*n* = 10); B: 4.8% (*n* = 6); *p*: 0.028). A comparison of long-term survival using Kaplan-Meier curves is given in Figure 2. Significant differences occurred between the groups. In contrast to a mean survival of 7.1 years in Group B (IQR 6.7–7.8 y), mean survival was reduced to 3.5 years (IQR 2.6–4.5 y) in the malperfusion group (log rank, *p* < .001). The outcomes for existing preoperative malperfusion are limited, especially during the early postoperative phase.

**Table 5.** Follow up data.

Characteristics	Entire cohort	Pre operative malperfusion	Non pre operative malperfusion	*p*-value
Total patients	*n* = 200	*n* = 74	*n* = 126	
Secondary aortic operation, *n* (%)	16 (8.0)	10 (13.5)	6 (4.8)	**.028**
Re-operation identic area, *n* (%)	5 (2.5)	4 (5.4)	1 (0.8)	.064
Re-operation downstream aorta, *n* (%)	11 (5.5)	6 (8.1)	5 (4.0)	.335
TAA repair, *n* (%)	2 (1.0)	0 (0.0)	2 (1.6)	.531
Y prothesis, *n* (%)	2 (1.0)	2 (2.7)	0 (0.0)	.136
Descending repair, *n* (%)	5 (2.5)	1 (1.4)	4 (3.2)	.653
Hybrid, *n* (%)	3 (1.5)	2 (2.7)	1 (0.8)	.556
Tevar, *n* (%)	3 (1.5)	2 (2.7)	1 (0.8)	.556
Evar, *n* (%)	3 (1.5)	2 (2.7)	1 (0.8)	.556
Aortic fenestration, *n* (%)	1 (0.5)	0 (0.0)	1 (0.8)	1.000

TAA thoracoabdominal repair, TEVAR thoracic endovascular aortic repair, EVAR endovascular aneurysm repair.

### Lactate levels

Levels of serum lactate were positively correlated with preoperative existing malperfusion. All measured lactate levels from the preoperative period until days 2–4 after surgery were significantly increased in the malperfusion cohort at all times. For detailed information see Table 6.

**Table 6.** Serum lactate levels in mmol/l (millimole per liter) over time. Preoperative, intraoperative, 24 hours after surgery, day 2–4 after surge.

Total patients	*n* = 200	*n* = 74	*n* = 126	*p*
Lactat max. preoperative mmol/l, median (IQR)	1.5 (0.9–2.4)	2.2 (1.4–3.2)	1.2 (0.8–1.9)	**<.001**
B (W) -lactat max. Intraop mmol/l, median (IQR)	5.3 (3.3–7.9)	6.5 (4.1–10.1)	4.5 (2.7–7.1)	**<.001**
Lactate max. 24 h after surgery mmol/l, median (IQR)	6.0 (3.5–10.6)	7.5 (4.7–12.8)	5.3 (3.2–8.9)	**<.001**
Lactate max d2-d4 mmol/l, median (IQR)	3.7 (2.4–6.2)	4.8 (3.0–8.5)	3.1 (2.2–5.3)	**<.001**

## Discussion

Acute type A aortic dissection is a manifold and life-threating disease. In the present study we distinguished patients with ATAAD regarding the existence (Group A) or absence (Group B) of preoperative malperfusion. Furthermore, the relevance of serum lactate levels as a marker for malperfusion was evaluated. The opinion that malperfusion is an independent risk factor is well established and evidence-based.^14–16^ Furthermore, Bennet et al.^17^ assume a correlation between increased serum lactate levels and one-year mortality. However, a simple assimilation of elevated lactate levels with malperfusion would be fatal. Hyperlactemia may also be caused by hypoperfusion during cardiogenic shock in a compromised cohort.^18^ However, is there an adjustment in perioperative management or surgical treatment due to proven malperfusion? And is serum lactate really a reliable marker for malperfusion during the decision-making process?

The patients’ conditions at admission to our hospital differed significantly between the groups. The malperfusion cohort fulfilled further independent risk factors,^19^ such as an increased incidence of preoperative intubation (A: 14.9%; B: 2.4%; *p*: 0.001). A significantly shorter onset of pain to surgery time (A: 4.0%; B: 7.0%; *p*: 0.001) may be associated with the occurrence of more severe clinical symptoms. Apart from painful symptoms caused by the malperfusion itself, an increased rate of preoperative stroke (A: 18.9%; B: 3.2%; *p*: < .001) as well as a higher prevalence of neurologic symptoms (A: 55.4%; B: 4.0%; *p*: < .001) support this thesis.

In keeping with the higher number of dissected supra-aortic arteries with proven malperfusion, perioperative data show a significant longer median SACP time in Group A (A: 76 min (28.5–96.5 min); B: 51.5 min (19.0–93.3 min); *p*: 0.011). In addition, mean cardiopulmonary bypass time was prolonged in the malperfusion group (A: 257.7 min (±81 min); B: 244.7 min (±77.8 min); *p*: 0.263), which is known as a predictor for postoperative mortality and morbidity.^20^ According to our data, based on the surgeon’s decision, patients with evidence of cerebral, visceral, renal, or limb malperfusion received a balanced ratio of limited and/or extended arch surgery. In contrast to these results, Kazui et al.^21^ found that extended arch repair could be applied without increased perioperative risks.

Patients with preoperative existing malperfusion fulfilled all the criteria for a limited postoperative outcome compared to the control group without malperfusion. Median survival time (A: 169.0 d; B: 1667.5 d; *p*: <0.001), 30-day mortality (A: 35.1%; B: 11.1%; *p*: < .001), median ventilation time (A: 89.0 h; B: 39.0 h; *p*: 0.001), duration of ICU treatment (A: 5.0 d; B: 4.0 d; *p*: 0.048), evidence of stroke (CCT) (A: 43.2%; B: 16.7%; *p*: < .001), as well as an extended requirement for postoperative dialysis (A: 29.7%; B: 7.9%; *p*: < .001) all express the serious consequence of a compromised patient’s condition prior to surgery. Neurological complications in particular remain one of the top issues in aortic surgery. Therefore, one of our main focuses is still the prevention of neurological events. In this study we performed moderate hypothermia in both groups combined with SACP.^22^ Using near-infrared spectroscopy is also well-established in our clinic. Given the fact that the new-onset rate of postoperative stroke was comparable in both groups (A: 12.2%; B: 12.7%; *p*: 0.912) we assume these neurological disabilities were induced preoperatively.

Postoperative visible (remaining) dissections also occurred in Group B. A new-onset cerebral malperfusion showed in 11 patients, and a new impairment of the renal perfusion in three patients. Ether dissection membrane proceeded between the first CT scan and surgery, or even preoperatively. All patients with remaining cerebral malperfusion received further interventions for the re-establishment of perfusion.

Despite the fact that both groups received a balanced extent of surgical procedures, the malperfusion cohort showed a significantly higher requirement for secondary aortic surgery (A: 13.5%; B: 4.8%; *p*: 0.28). Given the fact that the first surgery was an emergency procedure in a severely compromised cohort, the main goal was the survival of these patients. Thus, performing extended arch surgery was neglected in many cases, based on the patients’ conditions and the surgeon’s decision. Remaining pathologies had to be addressed in the later ongoing course.

It is known that malperfusion is an evidence-based risk factor for an increased mortality rate^23^ and that lactate levels are a reliable predictor for mortality after the repair of ATAAD.^1^ Furthermore, in comparison to existing publications, our study monitored lactate levels from the preoperative period until 2–4 days after surgery. Elevated serum lactate levels were significant at all time points in the malperfusion group. According our data, even early-stage surgical intervention in patients with malperfusion results in increased serum lactate levels from the preoperative period until day 4 after surgery.

Results of long-term survival using Kaplan-Meier curves confirmed an increased mortality rate during the early postoperative phase. If malperfusion occurs, the chance of survival is significantly reduced during the first year after surgery. Mortality rate is comparable with the control group one year after surgery (Figure 2). These are resonable data due to the fact that either acute or remaining malperfusion results mainly in acute organ failure.

Despite the proven existence of malperfusion an adjustment of the surgical therapy has not been taken place. Accordingly, further evaluations of the surgical procedure is mandatory. Goldberg et al.^24^ evaluated the interesting option of a “Reperfusion First” - Concept using surgical approaches like central repair, fenestration and direct revascularization. Nevertheless, the prompt aortic repair remains the gold standard.

### Limitations

This is a retrospective study and thus it carries all the potential risks and biases linked to studies of this nature. Furthermore, the individual decisions regarding the surgical procedures were based on the surgeon’s experience. Between the years 2008 and 2018, a total of 19 surgeons performed the operative treatment of these patients. Surgical skill levels may vary in this cohort. Furthermore, the diagnosis of a low cardiac output syndrome was not really evaluated in patients with ATAAD. Preoperative echocardiography data are not available for this population with a high urgent requirement for surgery. Only a minority of the patients presented malperfusion of one isolated perfusion area. For the further assignment of elevated lactate levels, multicenter studies are necessary. Furthermore, a relevant number of patients that died prior to admission can be expected.

## Conclusion

In this study, we identified patients with preexisting malperfusion due to ATAAD as a severely compromised cohort with significantly increased chances of early mortality. This cohort prematurely presents strong clinical symptoms with the consequence of a reduced onset of pain to surgery time. In all cases of malperfusion, serum lactate levels were a reliable marker for inadequate perfusion from admission until day 4 after surgery. Despite this, early intervention survival in this cohort is limited. It can be assumed that survival is comparable in cases of malperfusion after surviving the vulnerable perioperative phase and the first year after surgery.

## Appendix

### Abbreviations

ATAAD: Acute type A aortic dissection
BMI: Body mass index
CABG: Coronary artery bypass graft
CCT: Cranial computer tomography
COPD: Chronic obstructive pulmonary disease
COPD: Chronic obstructive occlusion disease
CT: Computer tomography
ECMO: Extracorporeal membrane oxygenation
EVAR: endovascular aortic repair
FET: Frozen elephant trunk
HCA: Hypothermic circulatory arrest
IQR: Interquartile range
IRAD: International Registry of Acute Aortic Dissections
LCA: Left coronary artery
PVOD: Peripheral vascular occlusion disease
RCA: Right coronary artery
SACP: Selective antegrade cerebral protection
SACP: Selective antegrade cerebral perfusion
TAA: Thoracic aneurysm aortic repair
TEVAR: thoracic endovascular aortic repair
